# Cavernous Sinus Thrombosis in a Three-Year-Old Male

**DOI:** 10.7759/cureus.116900

**Published:** 2026-09-25

**Authors:** Christina Kim, Ethan Kelly, Jessica Wimberly, Kaitlin Bettencourt

**Affiliations:** 1 Emergency Medicine, Madigan Army Medical Center, Tacoma, USA

**Keywords:** cavernous sinus thrombosis, emergency medicine, ent, otolaryngology, paediatric otolaryngology, pediatrics, septic cavernous sinus thrombosis

## Abstract

Cavernous sinus thrombosis (CST) is a rare disorder that can follow head and neck infections such as acute sinusitis, peritonsillar abscess (PTA), otitis media, and mastoiditis. The clinical presentation is variable, making CST difficult to recognize at initial presentation. This case report presents a three-year-old otherwise healthy male who presented to the emergency department with two weeks of intermittent fever and rhinorrhea and one day of lethargy and left-sided ptosis. Evaluation and workup in the emergency department included computed tomography (CT) and magnetic resonance imaging (MRI), which revealed a right-sided PTA, pansinusitis, and CST with dural involvement. The patient underwent PTA drainage, bilateral ethmoidectomy, adenoidectomy, and maxillary antrostomy in the operating room. This report highlights the importance of recognizing this low-incidence, high-morbidity diagnosis in the younger pediatric population and initiating early intervention to improve patient outcomes.

## Introduction

Cavernous sinus thrombosis (CST) in the pediatric population is a rare condition with an overall incidence of 0.34-0.67 cases per 100,000 children per year. The incidence of CST has decreased due to the high utilization of antibiotics; however, morbidity and mortality remain high due to rapid progression and neurologic involvement [1].

The presentation of a CST is variable, and symptoms can be nonspecific. Headache and fever are the most common chief complaints; however, younger patients may not be able to describe their symptoms, making recognition in the acute setting difficult [2-4]. Early recognition and intervention, including supportive care, antimicrobial therapy, and/or surgical drainage, are important for avoiding complications such as airway obstruction, aspiration, hematogenous spread, hemorrhage, or septic necrosis. This report presents a case of atypical symptoms in a young pediatric patient and highlights the importance of early intervention to improve patient outcomes. 

## Case presentation

A three-year-old, previously healthy, fully immunized male, with no significant medical history, presented to the emergency department with fever and nasal drainage ongoing for two weeks. He had been seen at an urgent care 10 days after symptom onset, where he was diagnosed with a viral syndrome. Symptoms had been improving with conservative measures at home; however, the patient's fever returned along with lethargy and new left eye drooping. His parents took him to his primary pediatrician, where labs, including blood cultures, were obtained, and he was referred to the emergency department for further evaluation due to his concerning presentation.

Initial vital signs were notable for fever, tachycardia, and tachypnea. His left-sided ptosis had resolved at the time of ED evaluation, and his head, eyes, ears, nose, and throat (HEENT) examination was otherwise normal. There were no signs of meningismus or neurological deficits, but he appeared lethargic. Labs were notable for leukocytosis with a left shift and elevated inflammatory markers (Table 1). The patient was given an IV fluid bolus and antipyretics, and he was empirically started on ceftriaxone and vancomycin.

**Table 1:** Patient labs, including CBC with differential, chemistry profile, and inflammatory markers. CBC: complete blood count; CO_2_: carbon dioxide; Na: sodium; K: potassium; Cl: chloride; BUN: blood urea nitrogen; Cr: creatinine; ALT: alanine aminotransferase; AST; aspartate aminotransferase; CRP: C-reactive protein; ESR: erythrocyte sedimentation rate.

	Unit	Patient	Reference range
Complete blood count			
White blood cell	K/uL	28.6	5.0-15.5
Neutrophil	%	79.1	-
Lymphocyte	%	8.6	-
Monocyte	%	11.3	-
Hemoglobin	g/dL	11.0	11.5-13.5
Hematocrit	%	33.8	33-40
Platelet	K/uL	553	160-500
Chemistry profile			
Na	mmol/L	132	135-145
K	mmol/L	4.3	3.6-5.3
Cl	mmol/L	97	98-109
CO_2_	mmol/L	26	21-32
Anion gap	-	9	4-12
BUN	mg/dL	5	4-18
Cr	mg/dL	0.32	0.3-0.7
Glucose	mg/dL	122	65-120
AST	IU/L	21	5-40
ALT	IU/L	10	6-60
Total bilirubin	mg/dL	0.4	0.2-1.4
Inflammatory markers			
CRP	mg/dL	6.0	0-1.0
Procalcitonin	ng/mL	0.97	<0.5
ESR	mm/hr	99	0-10

Computed tomography (CT) imaging showed a right-sided peritonsillar abscess (PTA) that tracked superiorly, abutting against the cavernous sinus, with associated CST and pansinusitis (Figures 1, 2). After discussion with neurology and otolaryngology, a magnetic resonance venography (MRV) was obtained to further characterize the intracranial extent of the infection. MRV images showed a large right-sided PTA (2.5x1.7 cm) with extension into the right cavernous sinus with associated dural enhancement, sinus thrombosis, and osteomyelitis of the clivus bone (Figure 3).

**Figure 1:**
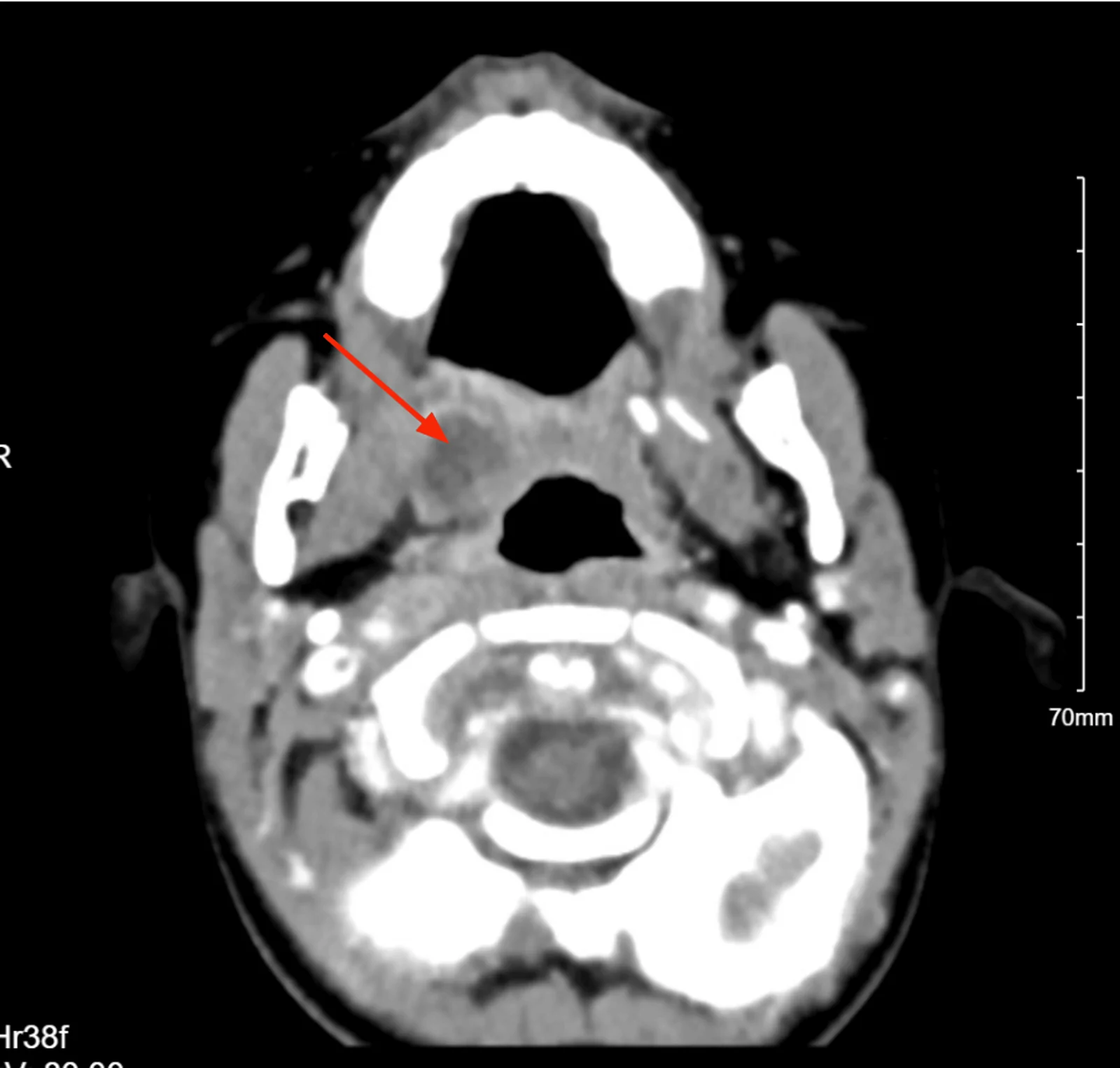
Axial view of contrast-enhanced maxillofacial CT demonstrating a hypodense PTA on the right (red arrow). CT: computed tomography; PTA: peritonsillar abscess.

**Figure 2:**
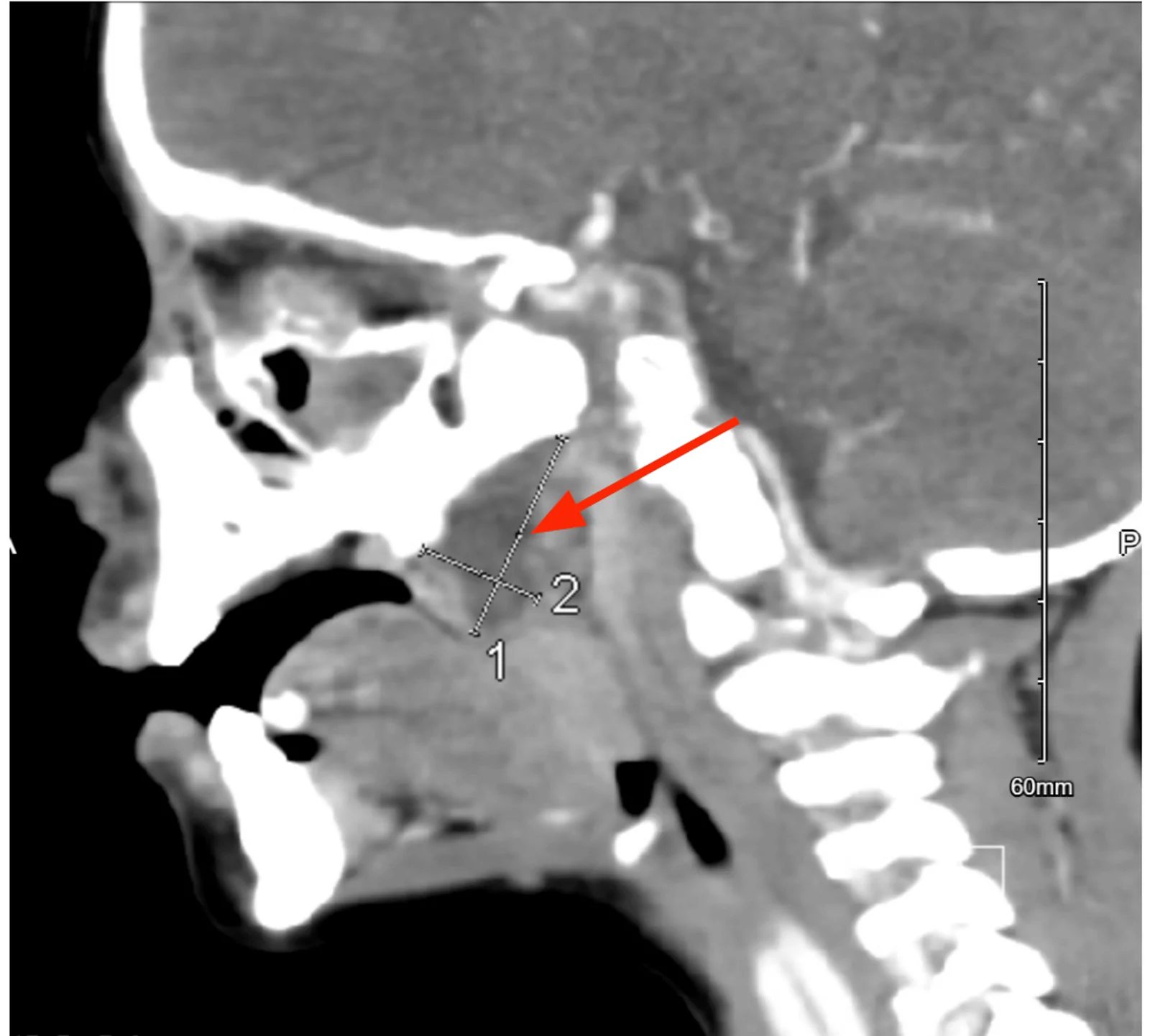
Sagittal view of contrast-enhanced maxillofacial CT redemonstrating the PTA on the right (red arrow). The numbers 1 and 2 indicate the length and width measurements, respectively. CT: computed tomography; PTA: peritonsillar abscess.

**Figure 3:**
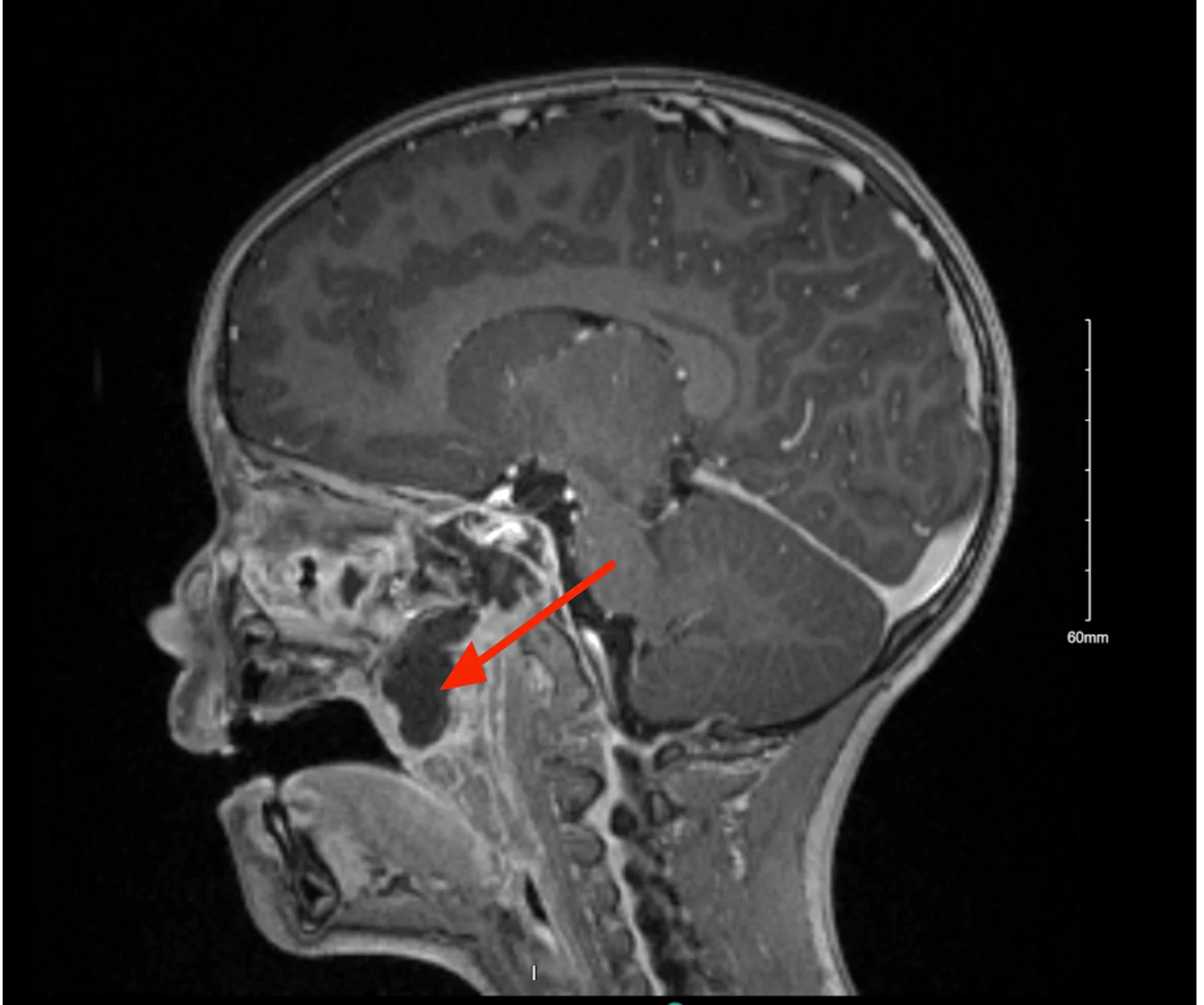
Sagittal view of non-contrast MRV of the head demonstrating a hypointense right-sided PTA (red arrow). MRV: magnetic resonance venography; PTA: peritonsillar abscess.

The patient was intubated for airway protection and taken to the operating room with otolaryngology, where he underwent PTA drainage, bilateral ethmoidectomy, bilateral sphenoidectomy, and maxillary antrostomy. He was admitted to the pediatric ICU for a week, where initial blood cultures grew Gram-positive cocci and PTA cultures grew *Staphylococcus intermedius*. His initial antibiotic therapy was broadened to include metronidazole for anaerobic coverage, and he was also started on therapeutic anticoagulation with heparin. He was downgraded to the pediatric floor after two weeks and was ultimately discharged neurologically intact.

## Discussion

Fevers and upper respiratory infections account for nearly 20-50% of all pediatric emergency department visits in the United States [5]. Most presentations are benign and do not require extensive workup or interventions; however, a high index of suspicion for a more severe pathology like CST is necessary when pediatric patients present with concerning histories and neurologic deficits.

Out of the dural venous sinuses, the cavernous sinus is the most frequently infected due to its central location within the intracranial venous system with multiple connections to adjacent structures and its complicated blood supply. Sinus, facial, dental, and otic infections have been associated with CST, with sphenoid and ethmoid sinusitis being the most common predisposing factors for CST [6].

Symptoms of a CST can be related to the structures that pass through the cavernous sinus. Patients may present with periorbital edema, proptosis, chemosis, cranial nerve III and VI palsies, and hyperesthesia of cranial nerve V. They may also complain of diplopia, photophobia, headache, and fever, with the latter two being the most common complaints. Recognition of CST in younger children is difficult, as they may not be able to voice their pain or symptoms.

Physical examination findings of cranial nerve palsies in a patient with a headache should make the clinician suspicious of a CST and consider diagnostic imaging promptly to prevent delay in treatment. The diagnostic imaging of choice is magnetic resonance imaging (MRI) with contrast and MRV, which have been found to be superior to CT in detecting thrombi in various stages. If MRI is not readily available, high-resolution orbital CT or CT venography of the brain can be considered as alternatives [7,8].

Prompt treatment for CST is necessary to avoid morbidity and mortality. Empiric antibiotics should cover staphylococci, including methicillin-resistant *S. aureus* (MRSA), streptococci, and anaerobic organisms [9]. Antifungals are not commonly initiated in the emergency department but should be considered in immunocompromised patients. The use of anticoagulants has been controversial but has been shown to improve morbidity and prevent bilateral extension in unilateral CST cases [10].

## Conclusions

CST is associated with significant morbidity and mortality, making early recognition and intervention extremely important. This disease typically starts as a facial infection, most commonly sinusitis, and can lead to symptoms that involve the specific structures within the cavernous sinus (cranial nerves III-VI). Clinical presentation can be highly variable and can be difficult to identify in the emergency department, especially in younger patients who may not be able to voice their symptoms. Patients with a headache, fever, and cranial nerve palsy on examination should make the clinician highly suspicious for a CST.

When CST is suspected, MRV is the diagnostic imaging study of choice, with a sensitivity approaching 90%. Empiric antibiotics should be initiated as soon as possible and should cover *Staphylococcus*, *Streptococcus*, and anaerobic species. Anticoagulants have not been shown to improve mortality but have been shown to reduce morbidity and should be considered as part of management. Patients with CST should be admitted to the hospital for continued antibiotics, anticoagulation, and possibly drainage or surgical intervention.
